# The *Corona mortis* is similar in size to the regular obturator artery, but is highly variable at the level of origin: an anatomical study

**DOI:** 10.1007/s12565-022-00671-w

**Published:** 2022-06-02

**Authors:** René Heichinger, Michael L. Pretterklieber, Niels Hammer, Bettina Pretterklieber

**Affiliations:** 1 Department of Orthopaedic and Trauma Surgery, Federal Hospital Zwettl, Zwettl, Austria; 2 Division of Macroscopic and Clinical Anatomy, Gottfried Schatz Research Center, Medical University of Graz, Harrachgasse 21/1 HG, 8010 Graz, Austria; 3 Division of Anatomy, Center for Anatomy and Cell Biology, Medical University of Vienna, Vienna, Austria; 4 Department of Trauma, Orthopedic and Plastic Surgery, University Hospital of Leipzig, Leipzig, Germany; 5 Division of Medical Technology, Fraunhofer Institute for Machine Tools and Forming Technology (Fraunhofer IWU), Dresden, Germany

**Keywords:** Arterial variation, *Corona mortis*, External iliac artery, Inferior epigastric artery, Obturator artery

## Abstract

An enlarged anastomosis connecting the vascular territory of the external iliac and the obturator artery may replace most or all of the latter. This relatively common vascular variation, known as *Corona mortis*, can lead to death in the worst-case scenario if injured. Despite being well-known, exact anthropometric data are lacking. The purpose of this study was to determine diameters of the regular obturator artery, the *Corona mortis* and the inferior epigastric artery. In addition, the level of origin of the *Corona mortis* was quantified. The obturator artery and its norm variants were dissected bilaterally in 75 specimens (37 females, 38 males) and measured using two different methods. The *Corona mortis* was present in 36 of the 150 hemipelves (24%), presenting in one third of all cases bilaterally. Its level of origin measured from the commencement of the inferior epigastric artery was subject to high variability (4.4–28.3 mm). The mean diameters of the *Corona mortis* (mean 2.5 and 2.1 mm, respectively) and the regular obturator artery (mean 2.4 and 2.0 mm, respectively) were similar for both methods. There were no significant sex nor side differences. The diameter of the inferior epigastric artery was significantly smaller distal to the origin of the *Corona mortis*. The high incidence, non-predictable level of origin of the *Corona mortis* and its size similar to the regular obturator artery support its clinical relevance even to date. Clinicians should always be aware of an additional arterial vessel close to the pelvic brim.

## Introduction

**Fig. 1:**
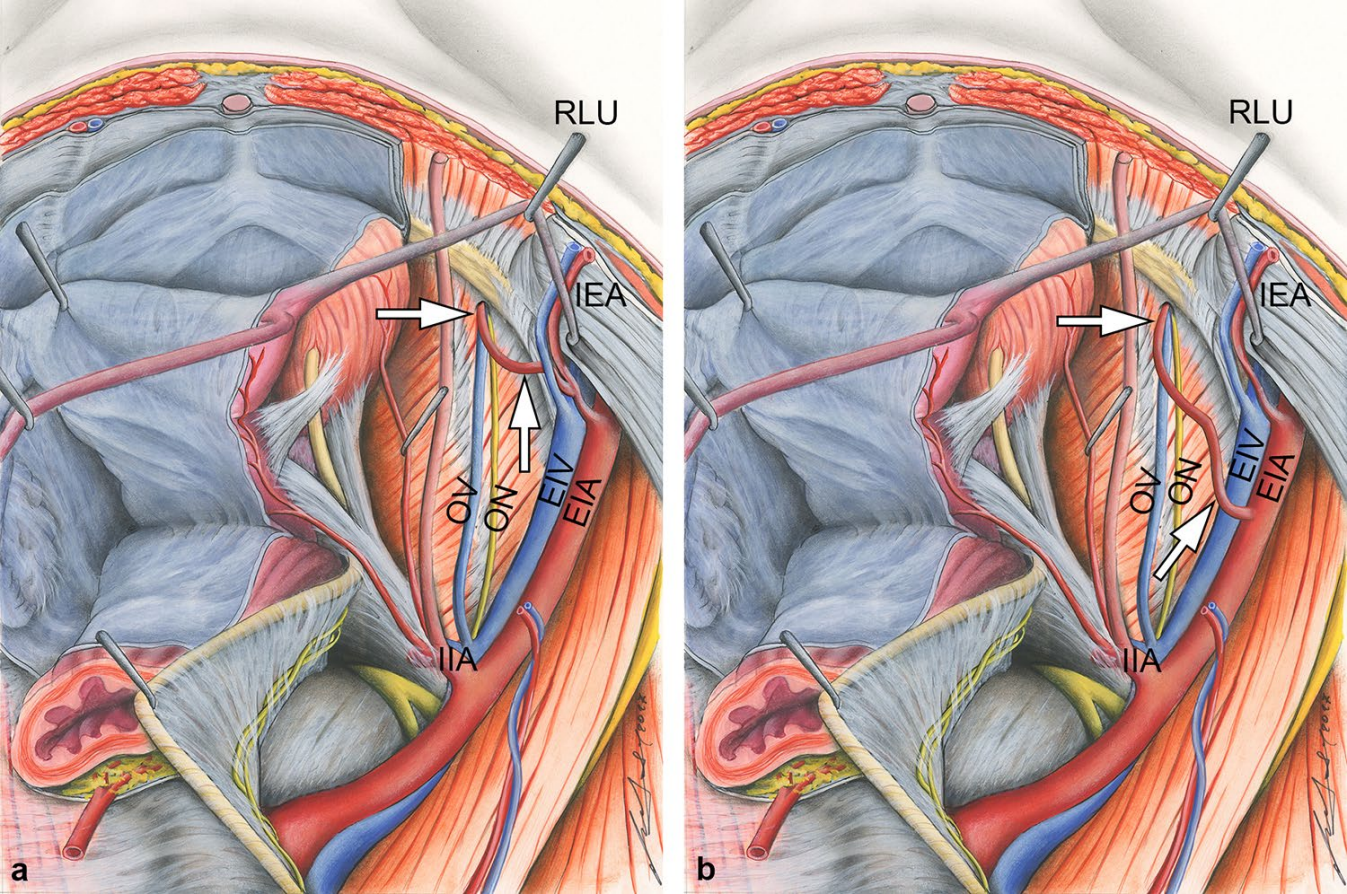
Two most common variants of a replaced obturator artery forming a *Corona mortis*. Most frequently, the *Corona mortis* (arrows) originates from the inferior epigastric artery (IEA) (**a**). In rare cases the *Corona mortis* (arrows) can arise directly from the external iliac artery (EIA) at a variable site within the greater pelvis (**b**). Dorso-cranial view into the lesser pelvis. In both examples, the *Corona mortis* joins the obturator nerve (ON) and vein (OV) before entering the obturator canal. *EIV* external iliac vein, *IIA* internal iliac artery, *RLU* round ligament of uterus

The obturator artery regularly originates from the internal iliac artery. Before entering the obturator canal, it gives rise to the pubic branch. This vessel anastomoses with the obturator branch of the inferior epigastric artery at the pecten pubis (Williams and Warwick 1980). In many cases, this anastomosis is a tiny vessel, but can exhibit a considerable larger diameter. In this case, it replaces all or most of the regular obturator artery, or uncommonly the inferior epigastric artery (Henle 1876; Luschka 1864; Engel 1859; Morris 1933; Gegenbaur 1888). This vessel is prone to injury during surgical repair of femoral hernias (Ates et al. 2016; Berberoĝlu et al. 2001; Lau and Lee 2003; Lipshutz 1918; Führer 1857; Jastschinski 1891; Merkel 1918; Hartmann 1881), or during gynaecologic, trauma, or orthopaedic procedures near the lateral wall of the lesser pelvis (Garrido-Gomez et al. 2012; Kong et al. 2012; Kacra et al. 2011; Darmanis et al. 2007; Stavropoulou-Deli and Anagnostopoulou 2013; Pellegrino et al. 2014; Perandini et al. 2018). Especially in former times, this replaced obturator artery turned out as a source of lethal bleedings. Thus, it was called *Corona mortis*, what means crown of death. The first author, who described the course of this replaced obturator artery was the anatomist Haller (1756) in the eighteenth century. In 1829 the surgeon Hesselbach (1829) was the first author, who used the old German term “Todenkranz”. Later, the Latin term “*Corona mortis*” was first found in Friedrich Führer’s “Handbuch der chirurgischen Anatomie” from 1857 (Führer 1857). In addition to this common variant of the obturator artery originating from the inferior epigastric artery, the former may also originate directly from the external iliac artery (Lippert and Pabst 1985). Both variants are sketched in Fig. 1.

The spatial location and the high risk of either traumatic or surgical injuries led to a high number of anatomical and radiological studies. These studies mainly focused on the incidence of a *Corona mortis* (Adachi 1928; Ates et al. 2016; Berberoĝlu et al. 2001; Braithwaite 1952; Cloquet 1817; Darmanis et al. 2007; Duenas-Garcia et al. 2017; Dwight 1894; Gilroy et al. 1997; Hartmann 1881; Hesselbach 1819; Hoffmann 1878; Jastschinski 1891; Karakurt et al. 2002; Kawai et al. 2008; Lau and Lee 2003; Lee et al. 2013; Lipshutz 1918; Okcu et al. 2004; Pai et al. 2009; Perandini et al. 2018; Pfitzner 1889; Pick et al. 1942; Quain 1844; Rajive and Pillay 2015; Schlobig 1844; Stavropoulou-Deli and Anagnostopoulou 2013; Steinberg et al. 2017; Teague et al. 1996; Tornetta et al. 1996; Wada et al. 2017; Al-Talalwah 2016). In light of techniques to prevent blood loss and minimally invasive exposures to hernia detailed knowledge of the *Corona mortis* is generally mandatory for surgeons. This is further important for trauma surgery especially in patients presenting with pelvic ring fractures. Therefore, data on the level of origin, and the diameter of a *Corona mortis* would be important orientation values for operations in this region. Only some authors performed measurements of the distance between the origin of the inferior epigastric artery and the origin of a *Corona mortis.* The data provided were either based on low numbers of cases (Lee et al. 2013), or there was no information given on the number of cases or mean values of the data collected (Henle 1876; Krause 1879; Luschka 1864). The diameter of the *Corona mortis* has been surveyed in only a few studies. The authors did not specify the measuring protocol nor did they compare their values with the diameter of a regular obturator artery (Teague et al. 1996; Tornetta et al. 1996; Okcu et al. 2004; Engel 1859).

We hypothesized that the calibre of the *Corona mortis* differs from that of the regular obturator artery. For this purpose, the diameters of both these variants were measured using a large number of embalmed anatomical specimens. Since it was additionally hypothesized that the level of origin of a C*orona mortis* from the inferior epigastric artery is constant, a further aim was to quantify the site of its origin. We also hypothesized that the diameter of the inferior epigastric artery should change significantly after the formation of a *Corona mortis* lacking previous evidence on this question.

## Materials and methods

This study is based on the dissection of 75 pelves of adult anatomic specimens of Caucasian origin (37 females, 38 males). While alive, all individuals bequested their post mortem bodies for their use in medical education and research. In addition to their informed consent, institutional approval was obtained from the Ethics Committee of the Medical University of Vienna (approval number 1221/2018). All specimens were embalmed by perfusion and immersion with a diluted solution of formalin (1.6%) and phenol (4%). Bodies with malignant tumours or vascular surgical interventions in the pelvic and femoral regions were excluded.

The frequency of a *Corona mortis* was determined overall, and regarding sex and body side. As generally accepted (Lippert and Pabst 1985), a *Corona mortis* was defined as follows: each vessel completely replacing a regular obturator artery, originating from the external iliac artery or one of its branches, and entering the obturator canal was called *Corona mortis*. In addition, connections between a regular obturator artery and the external iliac artery or one of its branches were defined as *Corona mortis* if the diameter from this anastomosis was the same or larger than that of the regular obturator artery arising from the internal iliac artery. In addition for this study, an inferior epigastric artery completely or partially originating from a regular obturator artery was also defined *Corona mortis*.

**Fig. 2:**
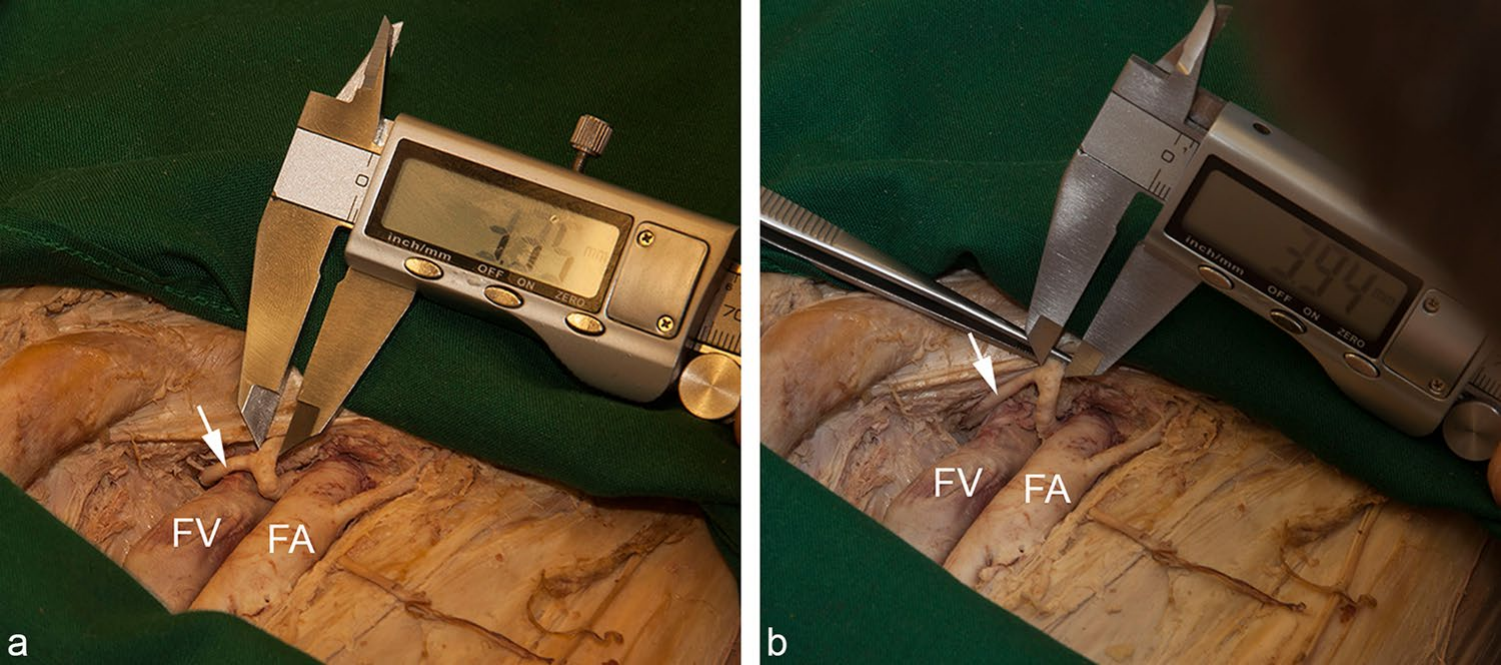
Methods of vascular diameter measurement. In this example, the inferior epigastric artery was measured within the vascular space distal to the exit of the *Corona mortis* (white arrow). With the method "unfolded", the outer diameter was measured without any compression of the vessel (**a**). Using the method "Hillen", the vessel was completely compressed with a forceps and thus half the circumference was determined. The diameter was then calculated from this value (**b**). *FA* femoral artery, *FV* femoral vein

To quantify the diameters of the regular obturator artery, a *Corona mortis*, and the inferior epigastric artery as well as the level of origin of a *Corona mortis*, measurements were conducted in millimetres rounded to one decimal place using a digital sliding calliper (Louisware, model LSWCL 1810, accuracy two decimal places). To minimize the probability of a methodological error, two different methods of measurements were performed. With the first one, the diameter of the vessels was determined without any compression. This method was named “unfolded” (Fig. 2a). For the second one, the vessel walls were compressed using an anatomical forceps. The diameter of this flattened vessel was assessed (Fig. 2b). This value corresponded with the half circumference of the vessel. Thus, the diameter was calculated with the formula: diameter = circumference/*π* ($d=\frac{c}{\pi }$). This method was established by Hillen (1986), so these results were termed “Hillen”.

**Fig. 3:**
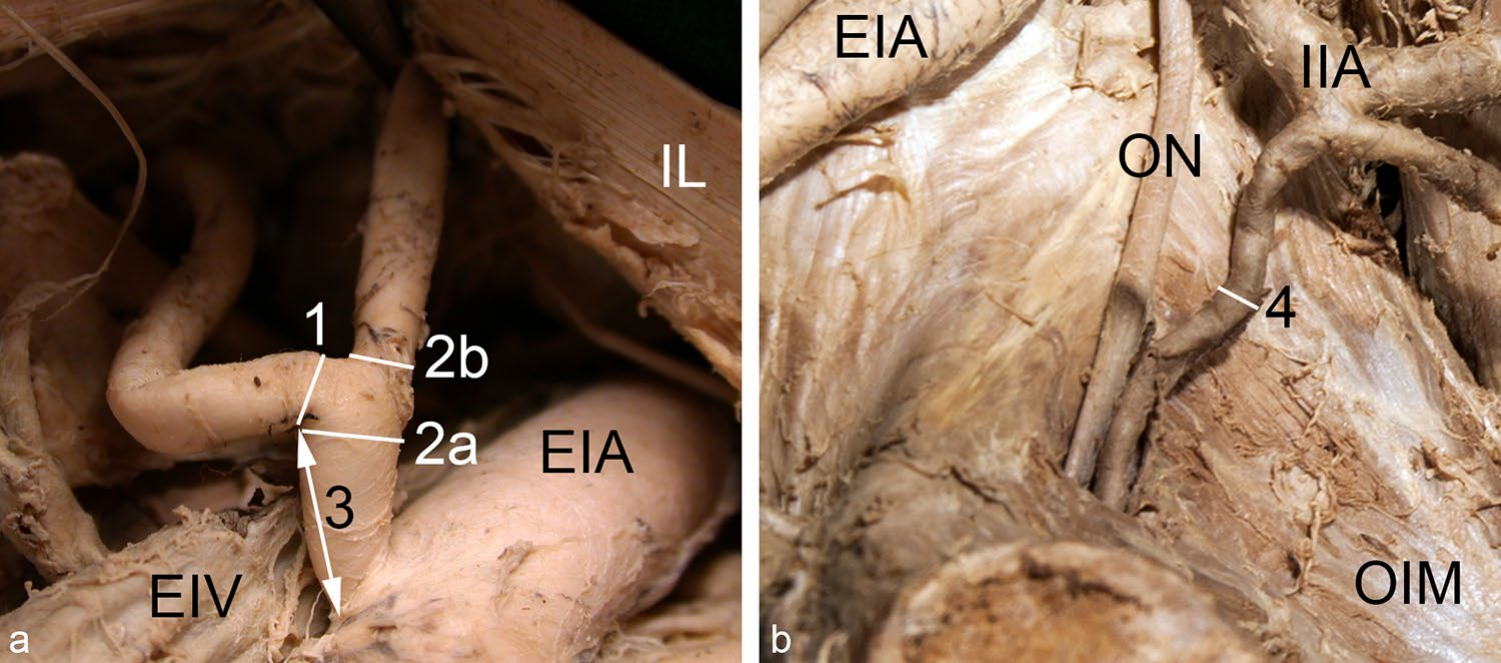
Measuring points for vessel diameter and level of origin. The diameter of the *Corona mortis* (1), the diameter of the inferior epigastric artery proximal (2a) and distal (2b) to the origin of the *Corona mortis*, and the distance between the origin of the inferior epigastric artery and the *Corona mortis* (3) were measured within the vascular space. View from distal (**a**). The diameter of the regular obturator artery (4) was measured half way between its origin and its entrance into the obturator canal. Wall of lesser pelvic, view from ventromedial, for a better overview the internal iliac vein and its branches have been removed (**b**). *EIA* external iliac artery, *EIV* external iliac vein, *IIA* internal iliac artery, *IL* inguinal ligament, *OIM* obturator internus muscle, *ON* obturator nerve

All measurements were conducted as follows (Fig. 3):

1. The diameter of the *Corona mortis* was measured directly at its origin.
2. The diameter of the inferior epigastric artery was measured directly proximal and distal to the origin of the *Corona mortis*.
3. To determine the level of origin, the distance between the origin of the *Corona mortis* and the origin of the inferior epigastric artery was measured at the closest points to each other.
4. The diameter of a regular obturator artery was measured in the middle between its origin and the entrance into the obturator canal. If the artery gave rise to a branch for the obturator internus muscle, then the measurement was performed distally to this branch to sustain the comparability to a *Corona mortis*. Only cases without an additional *Corona mortis* on the same side have been included.

Statistical analyses were performed using Excel (Microsoft Office Professional Plus 2016; Microsoft Corp., Redmond, WA, USA), IBM SPSS Statistics 26 (IBM Corp., Armonk, NY, USA), and GraphPad Prism 9.3.1 (GraphPad Software, San Diego, CA, USA). To prove a possible correlation between the incidence of a *Corona mortis* and sex or side of the body, a Chi-square test was used. The D’Agostino and Pearson normality test was used to assess Gaussians distribution of the data. Parametric data were then tested using an ordinary or repeated measures one-way ANOVA with Ŝídák’s multiple comparisons test, or a paired *T* test. Nonparametric data were tested using the Kruskal–Wallis test with Dunn’s multiple comparisons test, or the Wilcoxon matched pairs test.

The significance level was set at *α* = 0.05. In the text, mean values ± standard deviations are given.

## Results

### With an incidence of 24%, two-thirds of the *Coronae mortis* were unilateral with no sex nor side difference

A *Corona mortis* was observed in 27 individuals (36.0%). It was present in 16 of the 38 males (42.1%) and in 11 of the 37 females (29.7%). In relation to the pelvic halves, a *Corona mortis* occurred in 36 of 150 cases (24%). Thereby it was present in 19 male (25%) and 17 female hemipelves (23%).In the 27 individuals with a *Corona mortis*, it was present on only one side in 18 cases (66.7%). In the remaining nine individuals (33.3%) the *Corona mortis* was observed bilaterally. This was twice as much the case in females than in males (six vs. three individuals). In males, the unilateral cases were present in nine left and only four right sides. In females, the unilateral cases were more balanced (two left vs. three right sides). For detailed information, please refer to Table 1.

**Table 1:** Absolut and relative incidence of the regular obturator artery (OA) and the *Corona mortis* (CMOR) with regard to the pelvic halves

	Total (*n* = 150)	Female (*n* = 74)	Male (*n* = 76)	Right (*n* = 75)	Left (*n* = 75)
OA	114 (76.0%)	57 (77.0%)	57 (75.0%)	59 (78.7%)	55 (73.3%)
CMOR	34 (22.7%)	17 (23.0%)	17 (22.4%)	16 (21.3%)	18 (24.0%)
OA + CMOR	2 (1.3%)	0 (0.0%)	2 (2.6%)	0 (0.0%)	2 (2.7%)

There was no correlation between the occurrence of a *Corona mortis* with sex (*p* = 0.771) or with body side (*p* = 0.444).

**Fig. 4:**
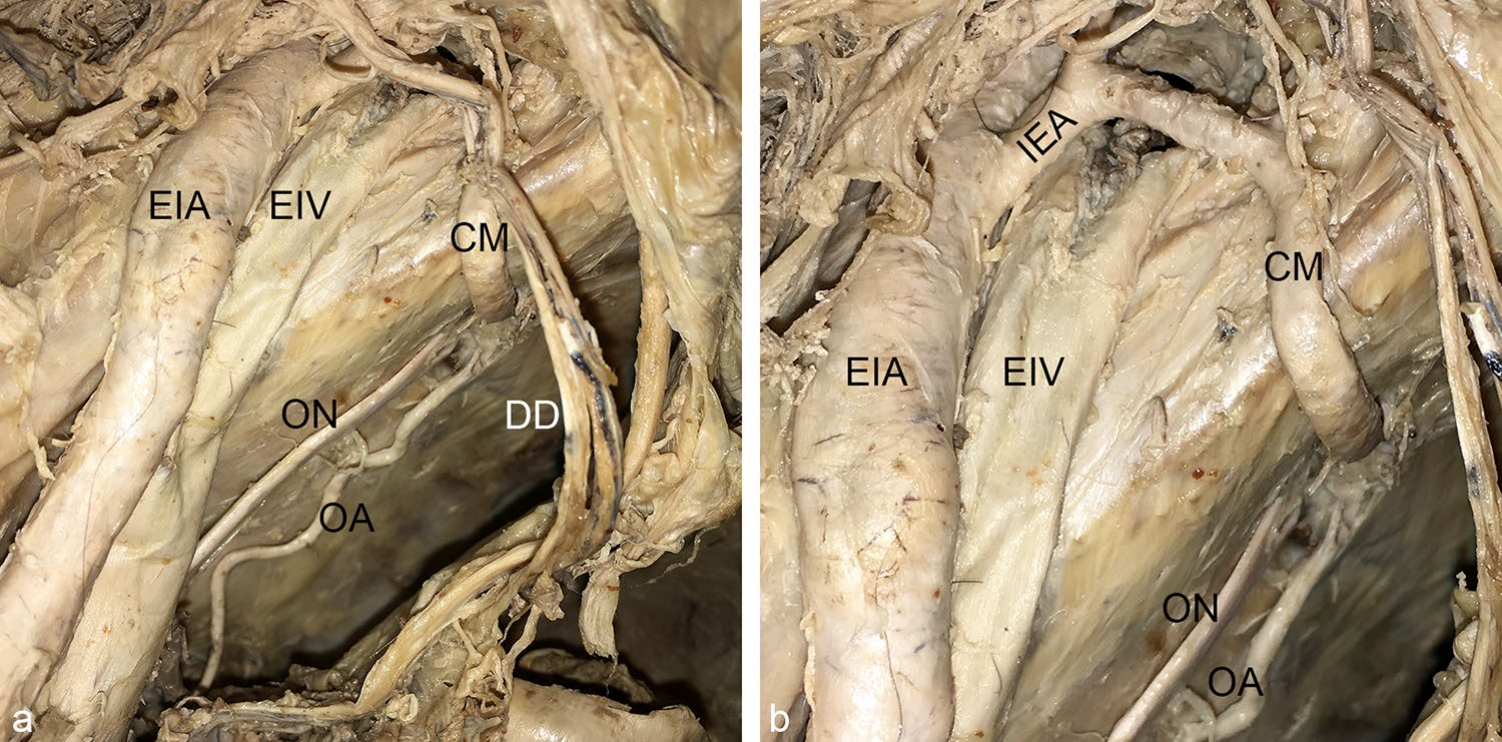
Co-incidence of a *Corona mortis* and a regular obturator artery. A *Corona mortis* (CM) and a regular obturator artery (OA) were present together in this left male pelvic hemisphere. Wall of lesser pelvic and vascular space, view from craniomedial, for a better overview the internal iliac vein and its branches have been removed, overview (**a**), detail (**b**). *DD* ductus deferens; *EIA* external iliac artery, *EIV* external iliac vein, *ON* obturator nerve

**Fig. 5:**
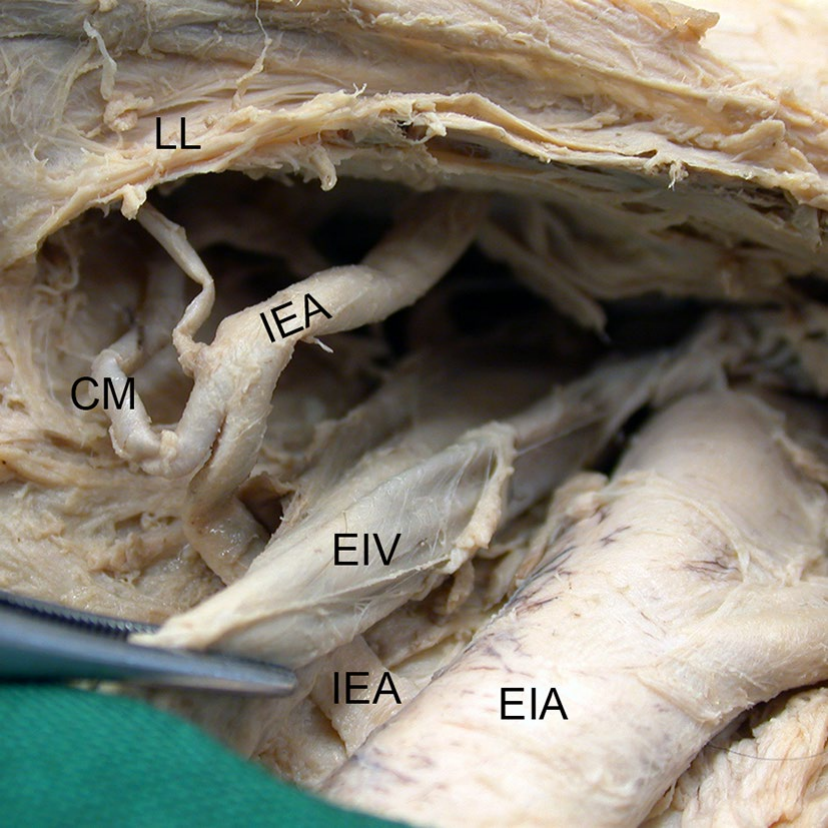
Reversed *Corona mortis*. In one left male hemipelvis, the *Corona mortis* (CM) partially replaced the inferior epigastric artery (IEA) instead of the obturator artery. Note the angle of junction and the greater diameter of the inferior epigastric artery (IEA) after the union with the *Corona mortis* (CM). Vascular space view from distal. *EIA* external iliac artery, *EIV* external iliac vein, *LL* lacunar ligament

A regular obturator artery together with a *Corona mortis* were coincidentally present in only two left male hemipelves (Fig. 4). In one of them, the *Corona mortis* did not partially replace the regular obturator artery but vice versa, the inferior epigastric artery. This could be concluded based on the angle of the junction with the inferior epigastric artery, and on the greater diameter of the latter after the junction with the *Corona mortis* (Fig. 5).

### High variability in the level of origin from the inferior epigastric artery without significant sex or side differences

**Fig. 6:**
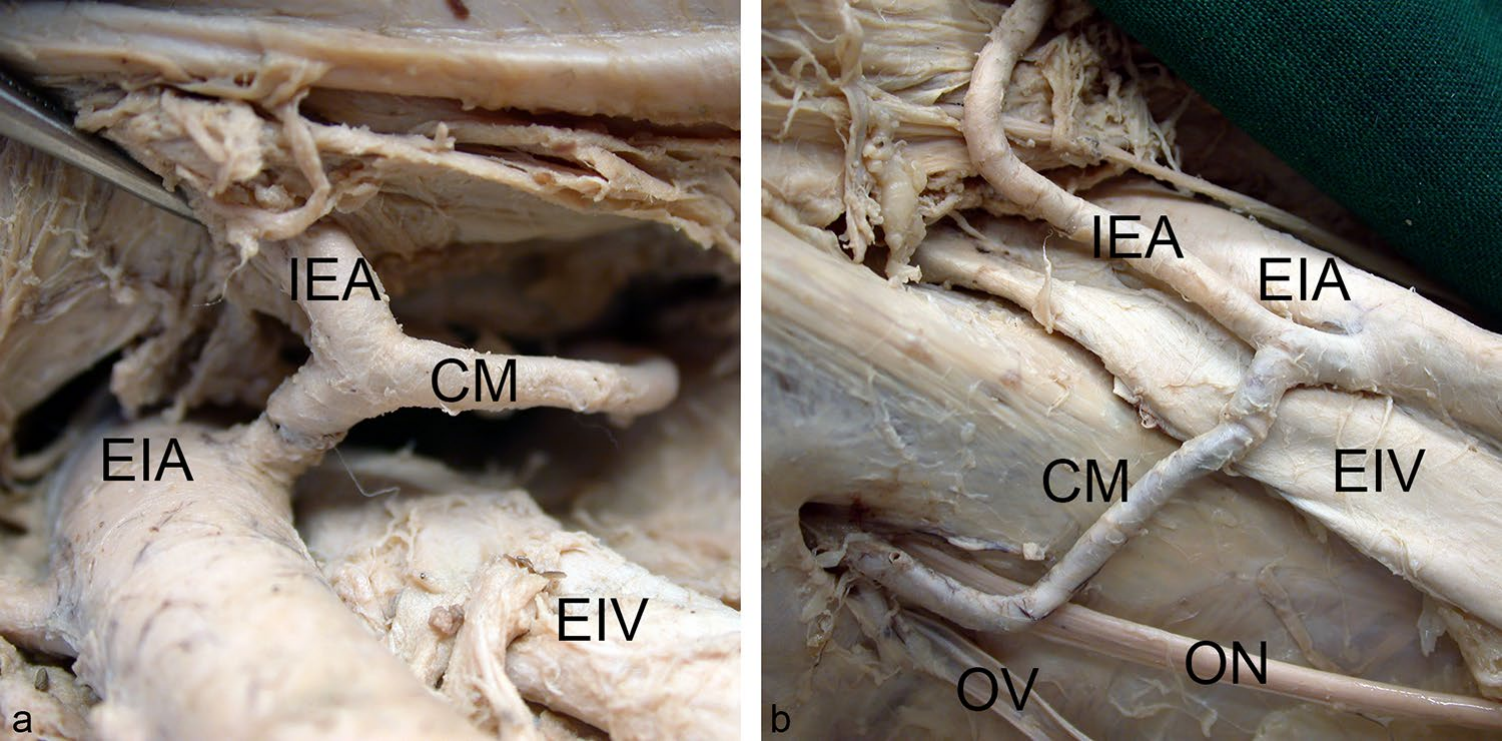
*Corona mortis* originating from inferior epigastric artery. An example of a *Corona mortis* (CM) originating from the inferior epigastric artery (IEA) within the vascular space. View from distal (**a**). An example of a *Corona mortis* (CM) originating from the inferior epigastric artery (IEA) proximal to the vascular space. Wall of lesser pelvic, view from medial (**b**). *EIA* external iliac artery, *EIV* external iliac vein, *ON* obturator nerve, *OV* obturator vein

**Fig. 7:**
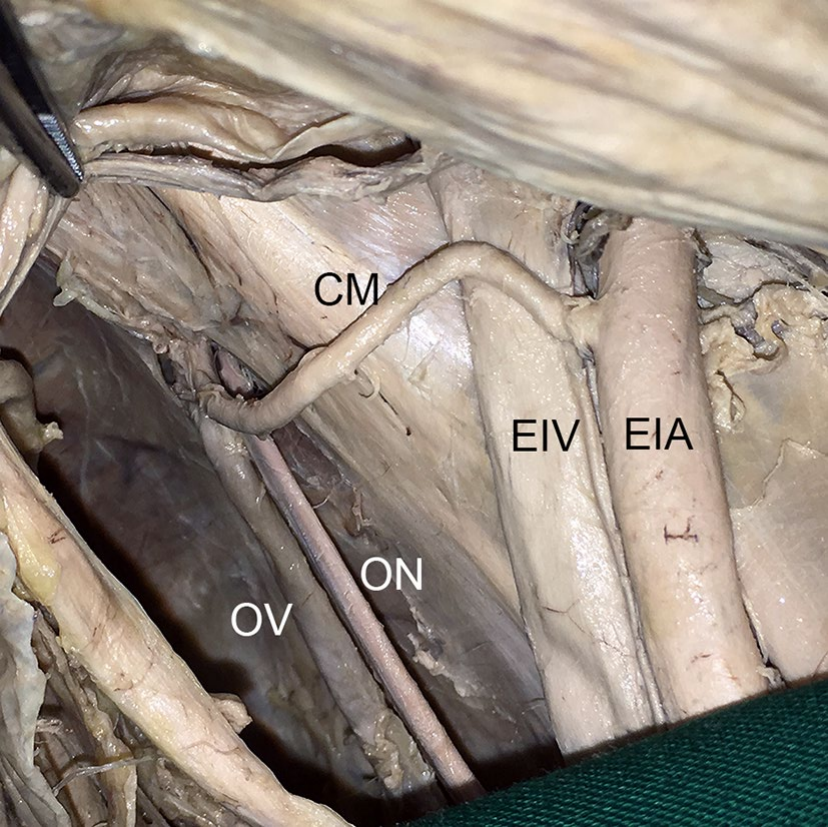
*Corona mortis* originating from external iliac artery. An example of a *Corona mortis* (CM) originating from the external iliac artery (EIA) proximal to the vascular space. Wall of lesser pelvic, view from craniomedial. *EIV* external iliac vein, *ON* obturator nerve, *OV* obturator vein

**Fig. 8:**
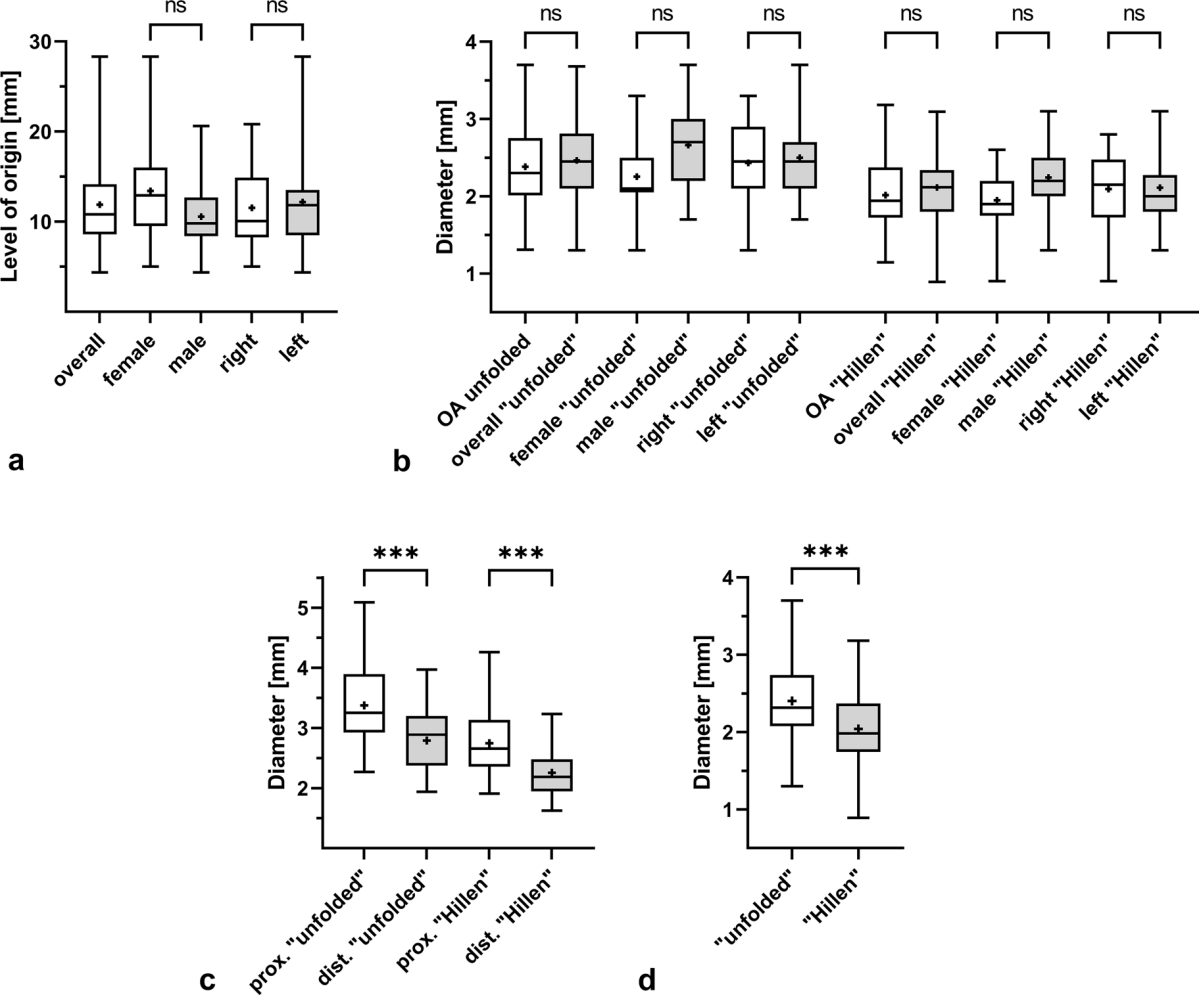
Boxplots summarizing levels of origin and diameters of the regular obturator artery, the *Corona mortis*, and the inferior epigastric artery. The level of origin of the *Corona mortis* from the inferior epigastric artery is depicted for all cases (*n* = 32), for females (*n* = 15) and males (*n* = 17), as well as for right (*n* = 14) and left (*n* = 18) sides (**a**). The diameters are given for the regular obturator artery (OA, *n* = 116) and for the *Corona mortis* overall (*n* = 36), in females (*n* = 17) and males (*n* = 19), as well as in the right (*n* = 16) and left (*n* = 20) side of the body. Thereby, the data obtained by the method “unfolded” are depicted within the left group, the data obtained by the method “Hillen” are depicted within the right group (**b**). The diameters of the inferior epigastric artery proximal and distal to the origin of the *Corona mortis* are presented for all cases (*n* = 32) and both methods (**c**). The diameters determined by the two methods “unfolded” and “Hillen” are given for all cases, regardless of whether a regular obturator artery or a corona mortis was present (**d**). The outlines of the boxes indicate the 25% and 75% percentile, the solid black line within the boxes represents the median. Whiskers indicate the minimum and maximum. *ns* not significant, ***p < 0.001

The *Corona mortis* originated in the majority of cases from the inferior epigastric artery (32 cases, 88.9%) (Fig. 6). In only four cases (11.1%), it arose from the external iliac artery (Fig. 7). The level of origin of the *Corona mortis* from the inferior epigastric artery was very variable (see Fig. 8a). On average, the *Corona mortis* originated 11.9 ± 5.0 mm (range 4.4–28.3 mm) distal to the commencement of the inferior epigastric artery from the external iliac artery. Although it originated more distally in women and on the left side on average, there was no difference between sexes (*p* = 0.210) nor sides (*p* > 0.999). In the four cases with *Corona mortis* origin from the external iliac artery, this was highly variable, ranging from 15.0 to 27.7 mm proximal to the commencement of the inferior epigastric artery. Due to the small number of cases, no further statistical analyses were conducted.

### No significant difference between diameters of *Corona mortis* and regular obturator artery

The diameter of the regular obturator artery averaged 2.4 ± 0.6 mm (“unfolded”) and 2.0 ± 0.5 mm (“Hillen”). This vessel originated in 79 cases from the ventral and in 35 cases from the dorsal trunk of the internal iliac artery. In two cases, it was impossible to identify a clear trunk-formation of the branches of the internal iliac artery. The average diameter of the *Corona mortis* was 2.5 ± 0.5 mm (“unfolded”), and 2.1 ± 0.4 mm (“Hillen”). The maximum diameter of the *Corona mortis* was 3.7 mm (“unfolded”) and 3.1 mm (“Hillen”). However, these values were measured in two different male individuals. The minimum diameter was determined in one female individual with 1.3 mm (“unfolded”) and 0.9 mm (“Hillen”). In cases with bilateral *Coronae mortis* the diameters were mostly the same on both sides and showed differences of 0.0 mm to at most 0.6 mm (both measurements methods). Only in one bilateral case, the difference amounts 1.2 mm (“unfolded”). For further details regarding sex and body side, please refer to Fig. 8b. There was no difference between the diameters of the regular obturator artery and the *Corona mortis*, neither for the method “unfolded” (*p* = 0.947) nor for the method “Hillen” (*p* = 0.880). There were also no statistically significant differences in the diameters of the *Corona mortis* between sexes or body sides for both methods.

### Diameter of the inferior epigastric artery is significantly smaller after commencement of the *Corona mortis*

The average diameter of the inferior epigastric artery proximally to the origin of the *Corona mortis* was 3.4 ± 0.7 mm (“unfolded”) and 2.8 ± 0.5 mm (“Hillen”). Distally to the commencement of the *Corona mortis* it was reduced to 2.8 ± 0.5 mm (“unfolded”) and 2.3 ± 0.4 mm (“Hillen”), respectively (Fig. 8c). With the exception of the case with the inverse *Corona mortis*, diameters of the inferior epigastric artery measured distal to the origin of the *Corona mortis* were significantly smaller from those measured proximally to its origin. This is true for the method “unfolded” (*p* < 0.001) as well as for the method “Hillen” (*p* < 0.001).

### Measurement methods provided significant differences, but measured values correlated highly between them

Diameters of the regular obturator artery and the *Corona mortis* measured with the method “Hillen” were significantly smaller than those measured with the method “unfolded” (*p* < 0.001). The mean difference was − 0.4 ± 0.2 mm (Fig. 8d). However, the diameters correlated highly between the two methods (*r* = 0.904, *p* < 0.001, *n* = 150).

## Discussion

### The high incidence of *Corona mortis* cases makes this anatomical variation a continued focus in anatomical–surgical research

To compare our findings on the obturator artery replaced by a *Corona mortis*, the incidence was calculated from 32 former anatomical, surgical, and radiological studies including 8.257 hemipelves (Table 2). The incidence of a replaced obturator artery branching off the external iliac artery, the external epigastric artery, or the femoral artery averaged 25%. The present study confirms this value, as the *Corona mortis* was observed in 24% of all hemipelves. Differences between studies can possibly be explained by the fact that the studies are based on different ethnic groups and on different numbers of cases. For example, the lowest incidences of 8.3% are based on only 26 and 36 cases. Conversely, the studies with the highest incidences (42–43%) are also based on case numbers far below 100. Among others, Japanese authors observed by trend a lower occurrence of a *Corona mortis* (Adachi 1928; Kawai et al. 2008; Wada et al. 2017).

**Table 2:** Review of previous studies on the incidence of a C*orona mortis* in relation to pelvic halves and comparison with the result of the current study

	Incidence (%)	Cases	Ethnicity	Method
Cloquet (1817)	30.4	500	Caucasian	Dissection
Hesselbach (1819)	42.2	64	Caucasian	Dissection
Schlobig (1844)	30.4	112	Caucasian	Dissection
Quain (1844)	31.6	361	Caucasian	Dissection
Hoffmann (1878)	32.5	400	Caucasian	Dissection
Hartmann (1881)	18.9	180	Caucasian	Dissection
Pfitzner (1889)	35.1	242	Caucasian	Dissection
Jastschinski (1891)	30.0	1034	Caucasian	Dissection
Dwight (1894)	25.8	500	Caucasian	Dissection
Lipshutz (1918)	19.3	181	Caucasian	Dissection
Adachi (1928)	13.2	692	Asian	Dissection
Pick et al. (1942)	29.0	640	Caucasian	Dissection
Braithwaite (1952)	20.6	169	Caucasian	Dissection
Teague et al. (1996)	43.0	79	Caucasian	Dissection
Tornetta et al. (1996)	34.0	50	Caucasian	Dissection
Gilroy et al. (1997)	38.0 33.0	45 60	Caucasian Asian	Dissection
Berberoĝlu et al. (2001)	14.3 8.3	14 26	Caucasian	Dissection Surgical
Karakurt et al. (2002)	28.5	98	Caucasian	Radiological
Lau and Lee (2003)	22.0	141	Asian	Surgical
Okcu et al. (2004)	19.0	150	Caucasian	Dissection
Darmanis et al. (2007)	37.5	80	Caucasian	Dissection
Kawai et al. (2008)	13.5	709	Asian	Dissection
Pai et al. (2009)	21.0	98	Asian	dissection
Lee et al. (2013)	8.3	36	Asian	Dissection
Stavropoulou-Deli and Anagnostopoulou (2013)	11.4	70	Caucasian	Dissection
Ates et al. (2016)	28.4	398	Caucasian	Surgical
Rajive and Pillay (2015)	26.0	50	Asian	Dissection
Wada et al. (2017)	14.3	196	Asian	Radiological
Al-Talalwah (2016)	9.8	208	Caucasian	Dissection
Duenas-Garcia et al. (2017)	27.9	174	Caucasian	Radiological
Steinberg et al. (2017)	33.0	200	Caucasian	Radiological
Perandini et al. (2018)	30.0	300	Caucasian	Radiological
Total	25	8257		
Present study	24.0	150	Caucasian	Dissection

In the present study, no correlation between sex and the occurrence of a *Corona mortis* was seen. However, males were more likely to have a unilateral *Corona mortis*, than females. On the other hand, females presented more often bilateral cases of *Corona mortis*, which lead to a balanced ratio between the sexes regarding only hemipelves. Most former authors did not consider the sex of the individuals in analysing their findings. However, contrary to our data, in some previous studies, females presented more often a replaced obturator artery (Engel 1859; Hollstein 1865; Dwight 1894; Jastschinski 1891). Although there was no correlation of the occurrence of a *Corona mortis* with body side, it was more often present on the left side. Former authors observed also only small differences concerning the side of its occurrence (Adachi 1928; Lau and Lee 2003; Steinberg et al. 2017; Dwight 1894; Engel 1859; Hesselbach 1819; Pfitzner 1889; Quain 1844; Pai et al. 2009; Stavropoulou-Deli and Anagnostopoulou 2013; Schlobig 1844; Pellegrino et al. 2014). Apparently, a *Corona mortis* can be expected to be present on both sides at an equal frequency.

The reverse variant, namely, the origin of the inferior epigastric artery from a regular obturator artery, was also considered a *Corona mortis* in this study. This vascular variant does not differ in location from a regular *Corona mortis*. Therefore we assume an equal risk of injury. In our sample, we observed this only once. The case was clear, based on the angle of confluence with the inferior epigastric artery and on the fact that the diameter of the latter was larger after union with the *Corona mortis*. There are no incidence data for the variant of this reverse *Corona mortis*. Only earlier anatomists described it as a rare variant (Engel 1859; Luschka 1864; Morris 1933; Gegenbaur 1888; Henle 1876). Our observation is therefore likely to be an exceptional case. Previous and current data concerning the presence of a *Corona mortis* show that surgeons of various disciplines may be faced with this potentially dangerous vascular variant at any time. This may be both the case in elective surgery, e.g., in hernia repair, and in cases with traumatic disruption of the pelvic ring with subsequent damage to the vessels supplying the anterior body wall and acetabular region.

### High variability in the level of origin from the inferior epigastric artery without significant sex nor side difference makes its exact position unpredictable

The level of origin of the *Corona mortis* in relation to the origin of the inferior epigastric artery was determined in a high number of cases for the first time in the present study. Henle (Henle 1876) and Krause (Krause 1879) specified this distance to be 4–40 mm and 5–40 mm, respectively. Unfortunately, both authors did not present case numbers or mean values. In one recent study, this distance was also determined. However, the level of origin was measured in only three cases of *Corona mortis* (Lee et al. 2013). The current study shows a very high variability of the level of origin of the *Corona mortis* from the inferior epigastric artery. On average, it originated 11.9 ± 5.0 mm distally to the commencement of the latter, but this distance varied between 4.4 mm and 28.3 mm. Therefore, it is impossible to give a recommendation where exactly to expect a possible *Corona mortis* during surgery.

### No significant difference between diameters of *Corona mortis* and regular obturator artery means similar amount of blood loss in case of injury

In the present study, the mean diameter of the *Corona mortis* was 2.5 ± 0.5 mm (“unfolded”), and 2.1 ± 0.4 mm (“Hillen”). It was slightly higher in male than in female bodies, but was almost equally concerning the side of the body. The mean diameters measured by other authors ranged from 1.63 to 4.0 mm (Hong et al. 2004; Tornetta et al. 1996; Perandini et al. 2018; Steinberg et al. 2017; Stavropoulou-Deli and Anagnostopoulou 2013). Although it is difficult to compare these data due to the lack of details in the methodology, it can be said that our values here are roughly in the middle. A comparison between sexes was made here for the first time.

In this study, for the first time, not only the diameter of the *Corona mortis* but also the diameter of the regular obturator artery was measured for comparison. The difference between their two mean diameters was only 0.1 mm for both measurements methods. Therefore, the *Coronae mortis* observed here are likely to have almost completely replaced the non-existent regular obturator artery. In practical terms, this means that injury to the *Corona mortis* may cause a similar amount of blood loss as an injury to the regular obturator artery.

### Diameter of the inferior epigastric artery is significantly smaller after commencement of the *Corona mortis*

This study was also the first to compare the diameter of the inferior epigastric artery proximal and distal to the origin of the *Corona mortis*. As expected, there was a significant reduction in diameter after commencement of the *Corona mortis*. On the other hand, this means that in the case of a *Corona mortis*, the inferior epigastric artery in its area of origin, is considerably stronger than usual. In practice, this means that in the case of a *Corona mortis*, not only is there an increased risk of bleeding from it. Injury to the inferior epigastric artery proximal to the origin of the *Corona mortis* may also result into higher amount of blood loss than in cases with a regular obturator artery. In the case of arterial occlusive disease, a *Corona mortis* may also become an important link between the circulation territories of the external and internal iliac arteries. People with this vascular variant may be better able to compensate for occlusions in this area and may not become symptomatic or may become symptomatic later.

### Both measurement methods have minor weaknesses, but seem suitable for the purpose of this and similar studies

To minimize the probability of a systematic error, the measurement of diameters was accomplished in two different ways. The determined values of both methods were statistically significantly different from each other but the measured values correlated highly between the two methods. The results of both methods are affected by different factors. The measurement described by Hillen (1986) may be influenced by sclerosis and thrombosis. In contrast, the method “unfolded” can deviate because of collapsed vessels. Due to the high correlation between the values of both measurement methods, it can be assumed that both methods are not entirely precise, but each is coherent in itself. Therefore, both methods are suitable for comparing diameters of two vessels or of one vessel at different sites. To determine only absolute measured values, slight deviations from the real values may occur with both methods. Measurements of absolute values on anatomic specimens can only ever approximate the situation during life—independently of the measuring method used—because of the variable blood pressure and the variable narrowing and widening of vessels in the living. It would therefore be interesting to investigate these vessel diameters radiologically in living subjects.

## Conclusions

The current study confirms previous data on the incidence of a *Corona mortis*. However, it was quantified for the first time that the level of origin of the *Corona mortis* is highly variable in relation to the origin of the inferior epigastric artery. In addition, it was demonstrated for the first time that the diameter of a *Corona mortis* is nearly identical to that of a regular obturator artery. It was also shown that the diameter of the inferior epigastric artery decreased significantly after the commencement of the *Corona mortis*. All these results support the clinical relevance of this common vascular variant: It can be expected in about a quarter of all hemipelves. One cannot predict at which level the *Corona mortis* originates from the inferior epigastric artery or the external iliac artery. Therefore, clinicians should always be aware of the possibility of an additional arterial vessel in this area, which can lead to severe arterial bleeding in the event of an injury.
